# Factors Behind the Higher COVID-19 Risk in Diabetes: A Critical Review

**DOI:** 10.3389/fpubh.2021.591982

**Published:** 2021-07-07

**Authors:** Amany Magdy Beshbishy, Victor B. Oti, Diaa E. Hussein, Ibrahim F. Rehan, Oluyomi S. Adeyemi, Nallely Rivero-Perez, Adrian Zaragoza-Bastida, Muhammad Ajmal Shah, Khaled Abouelezz, Helal F. Hetta, Natália Cruz-Martins, Gaber El-Saber Batiha

**Affiliations:** ^1^National Research Center for Protozoan Diseases, Obihiro University of Agriculture and Veterinary Medicine, Obihiro, Japan; ^2^Department of Microbiology, Nasarawa State University, Keffi, Nigeria; ^3^Researcher, Department of Food Hygiene, Agricultural Research Center, Animal Health Research Institute, Port of Alexandria, Egypt; ^4^Department of Husbandry and Development of Animal Wealth, Faculty of Veterinary Medicine, Menofa University, Shebin Alkom, Egypt; ^5^Medicinal Biochemistry, Infectious Diseases, Nanomedicine & Toxicology Laboratory, Department of Biochemistry, Landmark University, Omu-Aran, Nigeria; ^6^Área Académica de Medicina Veterinaria y Zootecnia, Instituto de Ciencias Agropecuaria, Universidad Autónoma del Estado de Hidalgo, Tulancingo, Mexico; ^7^Department of Pharmacognosy, Faculty of Pharmaceutical Sciences, Government College University, Faisalabad, Pakistan; ^8^Department of Medical Microbiology and Immunology, Faculty of Medicine, Assiut University, Assiut, Egypt; ^9^Faculty of Medicine, University of Porto, Porto, Portugal; ^10^Institute for Research and Innovation in Health (i3S), University of Porto, Porto, Portugal; ^11^Laboratory of Neuropsychophysiology, Faculty of Psychology and Education Sciences, University of Porto, Porto, Portugal; ^12^Department of Pharmacology and Therapeutics, Faculty of Veterinary Medicine, Damanhour University, Damanhour, Egypt

**Keywords:** diabetes, COVID-19, SARS-CoV-2, comorbidities, mortality

## Abstract

Diabetes mellitus (DM) and coronavirus disease 2019 (COVID-19) are public health issues worldwide, and their comorbidities trigger the progress to severe disease and even death in such patients. Globally, DM has affected an estimated 9.3% adults, and as of April 18, 2021, the World Health Organization (WHO) has confirmed 141,727,940 COVID-19 confirmed cases. The virus is spread via droplets, aerosols, and direct touch with others. Numerous predictive factors have been linked to COVID-19 severity, including impaired immune response and increased inflammatory response, among others. Angiotensin receptor blockers and angiotensin converting enzyme 2 have also been identified as playing a boosting role in both susceptibility and severity to severe acute respiratory syndrome coronavirus 2 (SARS-CoV-2). Specifically, in DM patients, both their control and management during this pandemic is herculean as the restriction periods have markedly hampered the maintenance of means to control glycemia, hypertension, and neuroendocrine and kidney diseases. In addition, as a result of the underlyin cardio-metabolic and immunological disorders, DM patients are at a higher risk of developing the severe form of COVID-19 despite other comorbidities, such as hypertension, also potentially boosting the development of higher COVID-19 severity. However, even in non-DM patients, SARS-CoV-2 may also cause transient hyperglycemia through induction of insulin resistance and/or pancreatic β-cell injury. Therefore, a strict glucose monitoring of DM patients with COVID-19 is mandatory to prevent life-threatening complications.

## Introduction

### COVID-19 Disease: An Overview

Coronavirus disease 2019, named COVID-19, discovered in Wuhan, China, is being quickly transmitted to the worldwide population. The viral agent, known as severe acute respiratory syndrome coronavirus 2 (SARS-CoV-2) is capable of inducing severe to acute pneumonia, lethal lung failure, and even death in susceptible hosts (1). Genetically, this virus differs from the closely related SARS members and is classified into the group of *beta coronavirus* infecting humans (2). Despite having a negative impact on the respiratory tract, clinical features range from asymptomatic to moderate-to-serious or critical disease. Currently, the viral infection case fatality rate is <5%, but 15–18% of infected patients may develop the severe form of the disease, even becoming critically ill, and others may need mechanical ventilation and intensive care unit (ICU) admission (3).

Declared a worldwide pandemic on March 11, 2020, by the World Health Organization (WHO) (4), the SARS-CoV-2 infection triggers various clinical manifestations, including respiratory tract disorders, severe pneumonia, septic shock, and multiorgan failure (4). However, clinical data available so far reveal that children aged <10 years usually remain asymptomatic and has a low case fatality rate (5). The median incubation period is 4 days, ranging from 2 to 14 days maximum (5, 6). The viral agent is shown to be spread via droplets, aerosols, and through direct contact with infected individuals. Concerning the virus dissemination via droplets, it can happen if a respiratory droplet is generated when an infected individual sneezes or coughs, and it is ingested or inhaled by close people (about 6 feet away). Thus, an individual may become infected by mere touching of surfaces or objects that harbor the viral agent and subsequently touching the nose, mouth, or eyes (7). Increased viral loads have been reported as soon as the symptoms start to appear, which suggests that viral transmission is more likely to occur early in viral infection (8). The duration of viral shedding differs based on the disease severity. For instance, Liu et al. (9) report that mild symptoms were found in 90% of COVID-19 patients who show a negative virus RNA test with swabs from the nasopharyngeal region by day 10 post-onset. Meanwhile, for a long time, the test remained positive in all severe cases. A study by Zou et al. (8) found that the detected viral load in patients without symptoms was similar to those that show symptoms. Indeed, there is evidence of spread of the viral agent during the incubation period of patients without symptoms (10).

### Diabetes: An Overview

Diabetes mellitus (DM) is among the world's greatest causes of morbidity with a high anticipation of exponential increase in the coming decades (11). The condition has several macro and microvascular complications as its predictors, which negatively affect the patient's overall survival (12). Globally, DM has affected about 9.3% of adults, there is a likelihood of it skyrocketing to 10.2% by the year 2030, and it might reach 10.9% by the year 2045 (13). In the last three decades, China has seen DM rise 17-fold, giving it the controversial slogan the “Diabetes World Capital” (14). Several metabolic predictors have been listed as triggers for metabolic disorders (DM and obesity, inclusive), such as chronic inflammation, oxidative stress, subclinical atherosclerosis, endothelial dysfunction, insulin resistance, glucose metabolism, and changed lipid, among others (14).

## Diabetes and Infectious Diseases

**Figure 1:**
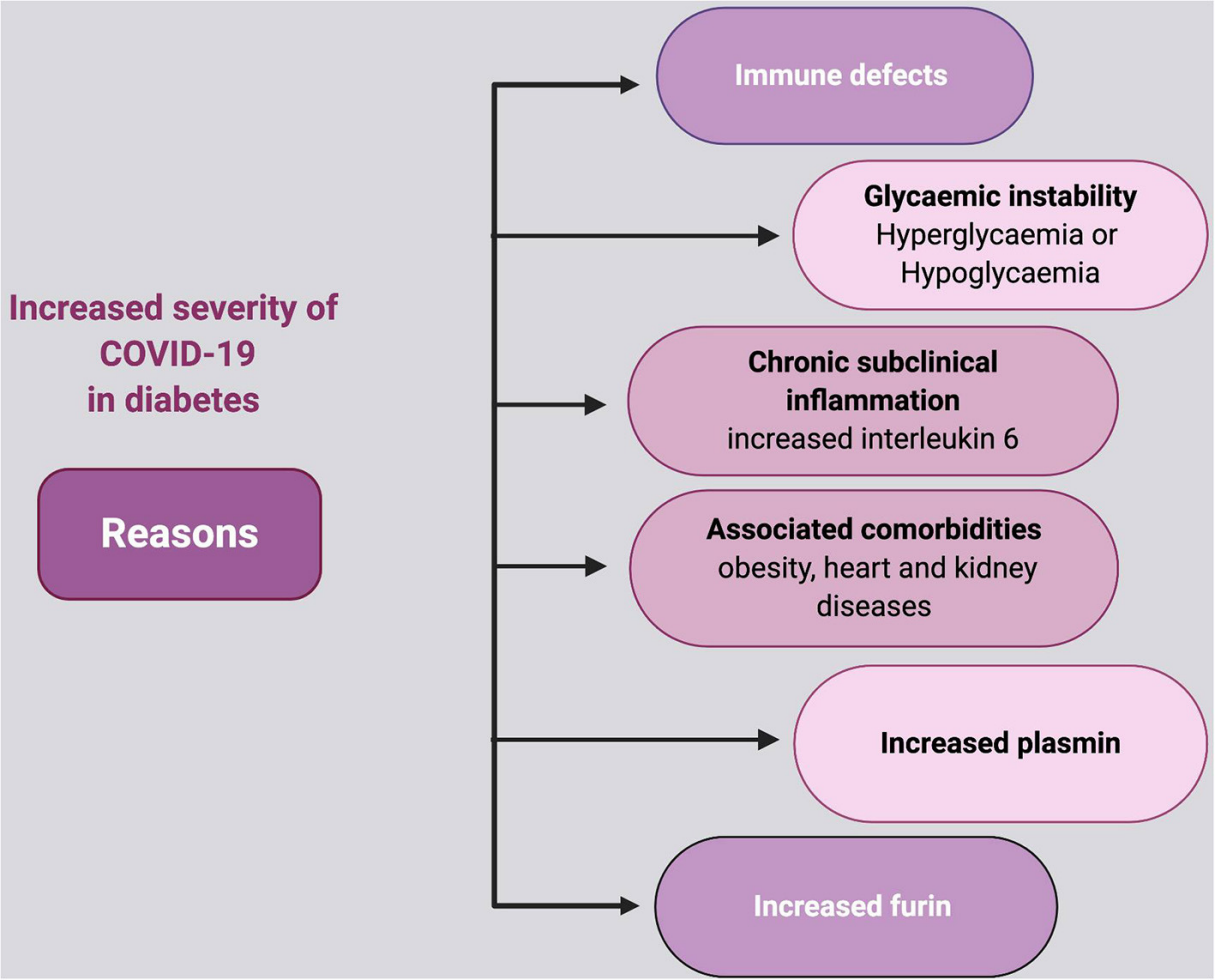
Viral entry mechanism to the host.

The interlink between DM and infectious diseases has been underlined by several clinical studies (15, 16). DM patients are more prone to infections as compared with those without DM as a result of impaired immunity (16, 17). Scientific evidence has found a greater rate of susceptibility to infectious diseases in DM individuals (Figure 1), such as those triggered by *Mycobacterium tuberculosis* and *Staphylococcus aureus* (18), directly attributed a dysregulated immune system (17). There is a general perception that DM patients can have higher morbidity and death rates by infectious diseases, and there is a paucity of epidemiologic data that would make it evident. There are reports that try to look at DM as a predisposing factor to some infection that can lead to death (19, 20). In this way, different viral infections are reported to increase the vulnerability of DM patients (21), such as those deriving from SARS-CoV (19), Middle East respiratory syndrome coronavirus (MERS-CoV) (21), and the 2009 influenza A (H1N1) infection (20). A recent review summarizing the clinical features linked to a higher risk of acquiring infectious diseases among DM individuals underlined that poorly controlled DM is a major risk factor, including for SARS-CoV-2 infection (22).

## Corona Virus Receptors

**Figure 2:**
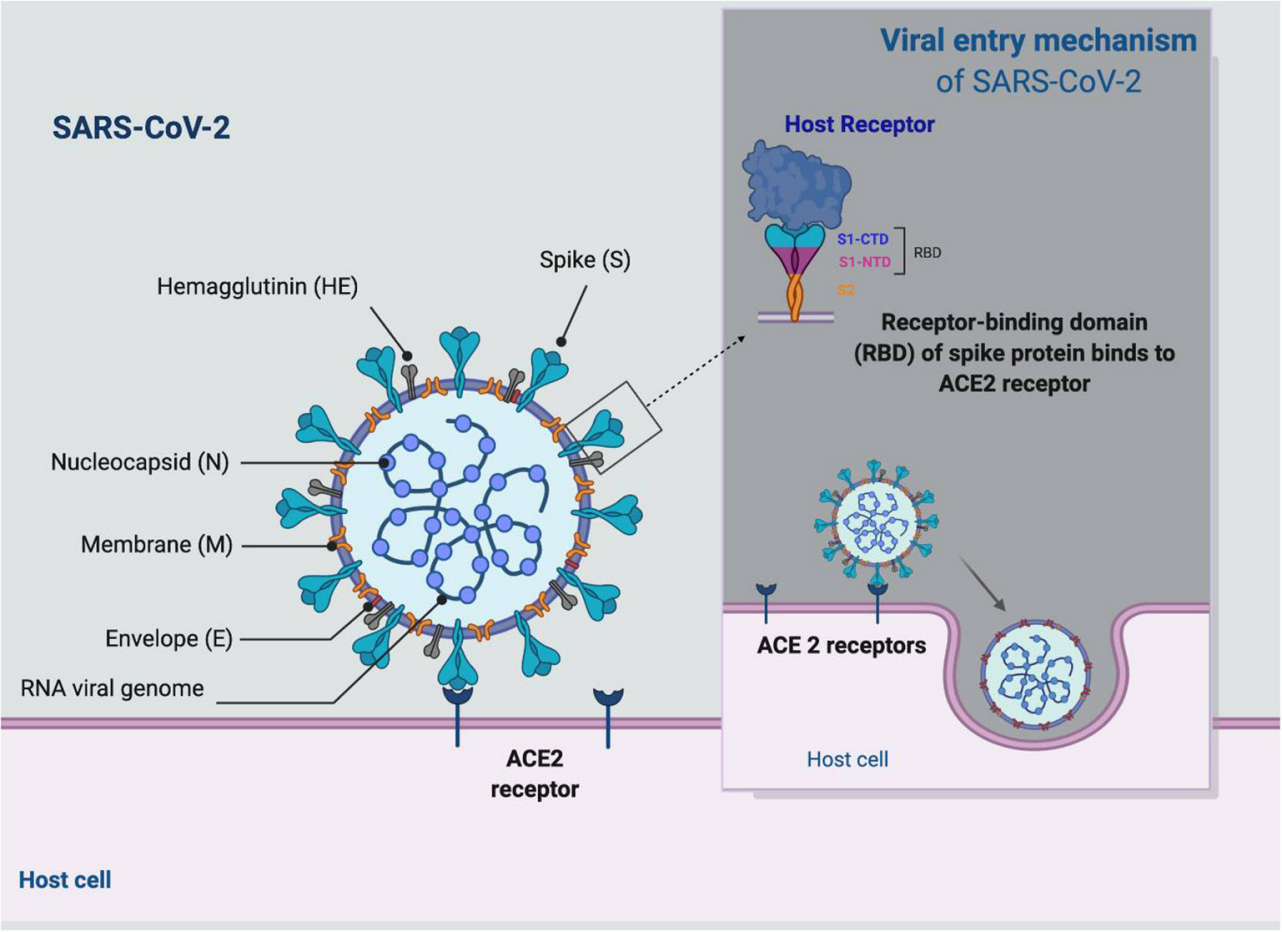
Diabetes as a risk factor for COVID-19.

SARS-CoV-2, similarly to SARS-CoV, uses angiotensin converting enzyme 2 (ACE2) as a cellular receptor (Figure 2), interacting directly with the spike glycoprotein (23). Wrapp et al. (24) report that the interaction between ACE2 and receptor-binding domain (RBD) is 10 to 20 times higher than the enzyme found in other coronaviruses. The enzyme has been found in myriad organ systems, including the heart, kidneys, brain, and lungs, among others (25). Indeed, there is evidence that the counter-regulatory enzyme ACE2, which metabolizes angiotensin II (Ang II), can enhance SARS-CoV-2 entry and replication (26). Many reports implicate the role of renin-angiotensin system (RAS) activation and reduced ACE2 expression in the process of lung injury following viral infection (27). As such, viral infection and type 2 DM (T2DM) have related pathogenic flow charts that have an implication in the treatment process (27). Additionally, dipeptidyl peptidase 4 (DPP4) and ACE2 regulate cardiovascular (CV) and renal physiology, inflammation, and glucose homeostasis. DPP4 inhibitors are greatly functional in T2DM patients due to their role in reducing glycemia although their role in the immune response of such patients is still not clear or fully established (28). Meta-analyses illustrate that DPP4 inhibitors do not raise the risk of acquiring infections (29–31), a feature that is also confirmed by Yang et al. (32), who did not report a high risk of infections due to pathogens with DPP4 inhibitors.

Indeed, a recent review highlights that deregulation of DPP4 receptors in COVID-19 as well as the upregulation of these receptors may be a determinant in COVID-19 severity. Therefore, the use of DPP4 inhibitors (gliptins) in COVID-19 management should be better exploited to improve the overall knowledge on how DPP4 may be useful to design novel therapies to block SARS-CoV-2 entry (33).

Other receptors on the human cell surface have been reported to facilitate the penetration of the viral agent, including transmembrane serine protease 2 (TMPRSS2) (34), extracellular matrix metalloproteinase inducer (35), and sialic acid receptors (36). Furthermore, cathepsins L and B have been reported to be key penetrating factors in the COVID-19 pathogenesis (34).

## Diabetogenic Viruses

Data from animal and human studies postulate that there are diabetogenic viral agents, but they are few (37). Two different studies found two DM patients who depend on acute insulin for a short and transient period following chicken pox infection (38). Guo et al. (26) recommend that viral infection could induce fast changes in blood glucose levels of DM patients, playing a negative role in such patients' recovery. Yang et al. (19) report an increase in fasting blood sugar (FBS), even in patients with non-severe SARS, who did not receive glucocorticoid therapy.

Briefly, coronavirus can trigger DM by inducing damage to the islets. This assertion is backed by the strong nature of immunostained ACE2 protein and its weak nature in exocrine tissues (39). Findings obtained so far show that DM and SARS-CoV-2 pneumonia can be entangled, and this may be detrimental to the COVID-19 prognosis.

## Morbidity of SARS-CoV-2 Infection in Diabetic Patients

Yang et al. (19) reveal that DM and plasma glucose levels are independent probable factors for morbimortality in SARS-CoV-2 infected patients. In China (Wuhan), a retrospective study showed that out of the 41 SARS-CoV-2 infected individuals, about 32% presented with other underlying diseases, 20% of them diabetic (40, 41). Research conducted in 8,910 patients with COVID-19 infection revealed that 14.3% had DM (42). Similarly, in a study performed in China with 1,099 hospitalized patients with novel coronavirus cases, 81 had DM, and about 34.6% had a serious ailment related to the 41/165 that had hypertension, 10/27 with coronary artery disorder, 67/261 with some coexisting disease, and 106/838 did not have any coexisting disease (43). Tentolouris et al. (44) carried out a study with 30,000 individuals in Greece and found a 6.6% incidence rate of DM. DM patients presented a higher incidence of comorbidities, including kidney, heart, and lung diseases. In addition, in the United States, CDC found that in 7,162 cases with incomplete data, above 50% of them were hospitalized, 10.9% had DM and 148 (18.9%) need admission into the intensive care unit (ICU) (45). The authors also found that the number of patients needing ICU admission did not differ from that with CV disease (20.4%) although it was higher than that with chronic lung disease (14.3%) or at an immuno-compromised status (15.5%). A report in Kuwait states that individuals with DM and obesity are more prone to ICU admission as the COVID-19 pandemic continues (46).

## DM: Risk Factor for COVID-19

Studies conducted in recent times show that the elderly or individuals with underlying comorbidities, such as CV disease, DM, and hypertension, are more susceptible to complications and possibly death as a result of COVID-19 (45). DM has also been found to be an important predictor for the severe form of COVID-19 due to respiratory tract infections (47). Also, DM has been reported to be linked to higher mortality following MERS-CoV and SARS-CoV infections by different studies (21, 47), and the level of FBS has been considered an independent probable factor for fatality in SARS infected patients (19). Diabetes is a possible predictor of infection, more so in those aged >60 years old and in those with poor glycemic control (48). Currently, there are no public findings, but obviously, there is a higher likelihood that DM patients will get SARS-CoV-2 infection (49). Moreover, it is known that DM and obesity are associated with the synthesis and clearance of dysregulated lipids that can trigger lung inflammation and injury (50, 51). In DM patients, the risk of pneumonia or lower and upper respiratory tract infections is greater than that of general population (52). Another study found that COVID-19 patients who have DM not only have a greater risk to severe pneumonia, but also spread inflammatory biomarkers excessively (25). However, DM patients might be at greater risk for hospitalization and development of complications due to COVID-19 (53).

Thus, the presence of patients with DM underlying viral infection is associated with two-fold heightened risk of death and also two-fold greater risk of COVID-19 severity (51). The main possible predictors include uncontrolled blood glucose levels before infection with the new coronavirus, shorter contact with DM healthcare experts, and unusual discontinuation of ACE2 inhibitor. The latter aspect mentioned is stated because reports show that the virus connects to cells via ACE2; therefore, these drugs can heighten the ACE2 level with the ability theoretically that there is a higher pickup of the viral agent. However, halting these medications can cause more damage in most cases (49). Indeed, related studies and reports have reported danger in DM individuals for the two new coronavirus infections discovered, SARS starts in 2002 in Asia and the Middle East, affecting more than 8,000 individuals (54) and MERS in 2012, mostly in Saudi Arabia, which affected more than 2,000 individuals (55).

## Comorbidity of Diabetes

DM is a comorbidity usually reported by COVID-19 patients (56, 57). Indeed, about 7% of COVID-19 patients have DM. Zhou et al. (47) state a recovery rate of 14% for COVID-19 patients with DM although a higher rate was stated (31%) for patients who were non-survivors. In a new published meta-analysis, it is found that DM incidence in ICU patients with viral infection increased twice in comparison with non-ICU patients (58). SARS-CoV-2 infection in DM patients can facilitate increased stress conditions with high release of hyperglycemic hormones, including catecholamines and glucocorticoids, ultimately resulting in high blood sugar levels and abnormalities in glucose levels (59). Recently, in the United States, the Centers for Disease Control and Prevention (CDC) reported that the incidence of DM was 10.9% in 7,162 patients who had all data on their health status on March 28, 2020 (58). Anyway, data published so far underline that DM patients and severe SARS-CoV-2 infection are older and have comorbidities when compared with non-DM patients (60).

## Mortality Rates of DM Patients with COVID-19

A recent study revealed that, among 191 Chinese COVID-19 patients hospitalized, 19% had DM with 54 deaths being recorded (45). The Chinese Center for Disease Control and Prevention (CCDC) registered a case fatality rate (CFR) of 2.3% in a detailed study with 44,672 COVID-19 patients. However, the rate increased to 10.5% in those with CV disease, 7.3% in those with DM, and 6.0% in those with hypertension (61). A fatality rate of 35% was also reported for those with DM as compared with 20% in the general population (62). Indeed, much research has reported that DM patients suffering from comorbidities have higher mortality rates due to complicated hyperglycemia and myocardial infarction because blood glucose plays a distinct role (63, 64). In addition, a recent multicenter retrospective study provides clinical evidence that correlate better blood glucose control with good clinical outcomes in COVID-19 patients and preexisting T2DM (65).

Regarding viral agent transmission, it occurs at a high efficiency, which impacts not only healthy individuals, but majorly the elderly, with higher complication rates than other pandemics (66). In several countries, these high rates of disease and death are often found in individuals of older ages and in those with comorbidities (67) although it is not certain if there are variations in the severity of infection between men and women with DM and if it differs between DMT1 and T2 (68). However, men are more prone to develop a severe form of COVID-19 due to underlying cardiometabolic risk factors, hormonal changes, ACE2 expression, inflammatory status, and exaggerated immune response (69). Also, Shahid et al. (70) illustrate that older subjects are at higher risk of death by COVID-19.

Additionally, a study shows that individuals who take corticosteroids for no obvious justification are at high risk (63). Thus, these patients are overrepresented in those at a high death risk from the virus: elderly people and those with comorbidities (5). Also worth noting is that it has been suggested that ACE2 can raise both viral infection susceptibility and severity through enzyme upregulation, which explains the overrepresentation of hypertensive patients in COVID-19 death causes (71). Finally, diabetic kidney disease (DKD) is also a leading factor that triggers disease and death in DM patients with estimations reporting that 30–40% of DM patients may die from DKD.

## Diabetes and Immune Response

Several abnormal immune system changes have been seen in DM patients, well-describing the relationship between immune dysfunction and hyperglycemia, involving weakening of the polymorphonuclear and monocytic white blood cell chemotaxis phagocytosis, complementary role and cytokine dysregulation (72). T2DM is generally recognized by a reduced level of chronic inflammatory disorder, triggered by a prolonged immune system imbalance, metabolic syndrome, and obesity (73). DM leads to proinflammatory homeostatic interactions of the immune system that is geared toward T helper 1 (Th1) and T17 cells and a decline in T-cell regulation (Treg) (17). Indeed, immune system dysfunction due to DM or infection has been observed in a variety of immune cells, not only CD4^+^ T cells, but also monocytes and macrophages (17). A recent study reported that the total number of CD8^+^ and CD4^+^ T cells lessens drastically and is functionally drained in SARS-CoV-2 infected patients, particularly in geriatric and in those critically ill who need ICU admission (74). Kulcsar et al. (75) found that DM mice had a long-term serious disease phase and postponed healing from MERS-CoV infection due to immune response dysregulation with lower rates of inflammatory CD4^+^ T cells and monocytes/macrophages. Moreover, several research studies found a greater risk of severe COVID-19 in DM patients with the increased risk of respiratory infections being linked to a compromised innate immune function. Nonetheless, temporary hyperglycemia may also partially alter the innate immune response to infection (76). Anyway, it is doubtless that DM patients are more prone to develop infectious diseases due to impaired immune system function (77). Moreover, DM patients with DKD have a chronic systemic inflammation resulting from a reduced immune system status that can lead to infectious complications, and together these may determine the risk of disease and death (78).

## Diabetes Complications

Increasing evidence states that DM-derived complications not only depict the disease severity, but also that such patients often have enhanced death rates that further justify the fact that DM is a key predictor in COVID-19 prognosis with DM severity being directly related to a bad prognosis (25). Reports also show that bad glycemic control, for example, evidenced by an increase in HbA1c, is importantly related to a higher risk to different infections (79, 80). Nonetheless, hyperosmolar hyperglycemic state (HHS) and DKA are still considered the most acute metabolic complications of DM, which are generally triggered by infection. Moreover, it is known that viral infection is tagged to precipitate the acute hyperglycemic issues in uncontrolled DM patients, but there is still a paucity of scientific evidence regarding this association (81). Zhang et al. (82) state that COVID-19 patients with DM are more prone to generate severe subtypes, including acute respiratory disease (ARDS) complications and acute cardiac injury that occurs due to mechanical ventilation and antibiotic treatment.

### Diabetes and Hypertension

Globally, hypertension is viewed as one of the most generalized diseases and comorbidities in DM patients and considered as a silent killer (83). Published 2020 studies from China's Wuhan province show that patients with hypertension and DM are overrepresented among the sickest COVID-19 patients and those susceptible to the disease (5, 63). The increased rate of hypertensive patients with SARS-CoV-2 is reported in a study with 41 patients found in Wuhan hospital (84) although a larger analysis of 138 hospitalized patients also report a similar outcome (40). Similarly, a study conducted in Jinyintan Hospital and Wuhan Pulmonary Hospital among 191 patients with the novel coronavirus found hypertension in 58 (30%) patients, 26 (48%) did not survive the viral infection, and 32 (23%) survived (45). Another study performed in Switzerland found that hypertensive patients (23.7%) with cerebrovascular disorders (22%) and diabetes (22%) were the most striking comorbidities (65). Recent findings also point out that the CV system is the second target system of the virus besides the lungs. Therefore, hypertension is linked to poor clinical outcomes in COVID-19 patients, and current ACEIs or ARBs should not be discontinued (85, 86).

### Diabetes and Endothelial Dysfunction

DM is typically characterized by endothelial dysfunction apart from hyperglycemia and microcirculatory impairment (87). More than one third of newly diagnosed T2DM patients may have microvascular disease (88). The bad blow of endothelial impairment involves fibrinolysis, vasodilation dysregulation, and anti-aggregation, which typically leads to macrovascular disorders (81). Nevertheless, the evidence of microvascular impairment in one organ is a key indicator of systemic damage. There is evidence that such complications derive from microvascular lung diseases of DM (“diabetic lung”) (89). Furthermore, atherosclerosis, endothelial impairment, and vascular inflammation are also involved in the pathogenicity of other chronic disorders, such as CV diseases and hypertension (90). Indeed, endothelial dysfunction in DM patients with COVID-19 might be due to direct invasion of vascular endothelial cells by SARS-CoV-2 through endothelial ACE2 (91). As well, the high levels of pro-inflammatory cytokines and the development of cytokine storm (CS) in severe COVID-19 patients may cause endothelial injury and boost the development of endothelial dysfunctions (92).

### Diabetes and Thrombosis

Coagulation dysfunction is also reported in severe COVID-19 patients (93) with fatal cases presenting diffuse microvascular thrombosis, which is suggestive of a thrombogenic microangiopathy (94), In T2DM, aside from the marked inflammatory stage, an imbalance also exists between coagulation and fibrinolysis and also enhances clotting factor levels and relative fibrinolytic system stoppage (72). T2DM and insulin resistance are known to be related to endothelial cell dysfunction and increased platelet activation and aggregation. These abnormal changes foster the generation of hypercoagulable pro-thrombosis. Guo et al. (25) posit that, during the inflammatory storm, the D-dimer rises geometrically. It is an outcome of inflammation activating plasmin during the early stage. However, as inflammation goes on in hypoxia-induced molecules, which can trigger direct thrombin and activate monocyte-macrophages, which can also emit tissue factors in masses and open the pathway of exogenous coagulation, this results in a general state of hypercoagulability. For example, Guo et al. (25) found that the level of FIB and D-dimer increased greatly in DM patients, which is an indicator of prone hypercoagulable state as compared to non-DM patients. McFadyen et al. (95) reveal that SARS-CoV-2-induced endothelial dysfunction may lead to immune-related thrombosis due to both platelet and complement activations so that the use of anti-thrombotic agents, such as heparin and dipyridamole, may prevent these complications in COVID-19 patients.

### Diabetes and Kidney Disease

Acute kidney injury (AKI) has been found in <20% of critically sick and COVID-19 patients with this finding being supported by data from China (63), Italy (96), and the United States (97). A study carried out in 2020 found that kidneys have an increased vulnerability to ACE2 expression-based destruction (98), and in addition, kidney disease is a common DM-related clinical complication. Worldwide, more than 2 million individuals receive dialysis therapy, and up to half a million individuals are receiving maintenance dialysis in the United States, which poses a greater danger for the acquisition of SARS-CoV-2 infection. The CDC and the American Society of Nephrology (ASN) have compiled best practices for in-center dialysis (99). Marked changes in immune system function have been stated in patients with kidney-related problems; nonetheless, despite immune system impairment, meticulous attention should be paid on the uremic state because the excessive oxidative stress status caused by excess toxin retention and gathering of synthesized products of oxidation can exacerbate the clinical status in infected patients (72). A study recorded that endothelial damage is normal in 26 patients with SARS-CoV-2 renal histopathology without interstitial inflammatory infiltration (100). However, so far, there are still not certain reports with respect to the kidneys. The direct mechanism in viral infection includes syndrome of CS via targeted viral renal tubular cell destruction, and sepsis pathways have been investigated (101). Presently, the major expression of renal destruction in SARS-CoV-2-infected patients looks to be acute although certain conditions of proteinuria/hematuria and macroalbuminuria may be interrelated to endothelial cell impairment found in such patients (102).

#### Chest Tomography (CT) Imaging and Biochemical Tests of Diabetic Patients

In DM patients, a chest CT scan usually depicts ground-glass opacifications with abnormalities that are united or not. Involvement in normal lobes can also be seen bilaterally with peripheral features of COVID-19 (103). Moreover, although normal CT images can also be found in some confirmed cases (104), abnormal images have also been seen in asymptomatic patients. For instance, Guo et al. (25) report that the DM patients' group had an increase in the CT image score when compared with those non-DM who have COVID-19 pneumonia with clinical status being also more serious than in those without DM. Into the bargain, the retrospective study by Wang et al. (105), involving 307 COVID-19 patients, shows that early definitive diagnosis could be grasped by age-related clinical and CT scan findings. Moreover, lung CT scan findings were correlated with COVID-19 severity and development of acute ischemic stroke in T2DM patients with COVID-19 (106).

Regarding biochemical outcomes in such patients, some authors have already stated that few enzymes serving as indicators are raised aberrantly in the blood of viral pneumonia patients, including α-hydroxybutyrate dehydrogenase (HBDH), gamma glutamyltransferase (GGT), lactate dehydrogenase (LDH), and alanine aminotransferase (ALT), which shows myocardium, liver, and kidney injury. This outcome is in consonance with the excessive frequency of viral receptors (ACE2) and may partly explain why death occurred in some patients with multiple organ failure (107, 108).

#### Blood Picture of Diabetic Patients With COVID-19

SARS-CoV-2-infected patients commonly depict lymphocytopenia on entry and, to a limited degree, leukopenia and thrombocytopenia, more conspicuous in those with severe disease status (5). Elevated pro-inflammatory cytokine levels, which include, among others, C-reactive protein (CRP), interleukin (IL)-6, and enhanced coagulation activity, shown by higher d-dimer concentrations, have also been correlated with disease severity (45). Furthermore, DM patients had greater neutrophils, IL-6, white cells, LDH, IL-2R, CRP, IL-8, D-dimer, and N-terminal pro-B-type natriuretic peptide (NT-proBNP) levels and lower lymphocyte counts. Taken together, these outcomes indicate an increased response to pro-inflammatory cytokines compared with non-DM patients (58). Moreover, the serum levels of some inflammation-related biomarkers are higher in DM when compared with non-DM individuals, such as IL-6, ESR, CRP, and serum ferritin. Additionally, a pronounced raise in serum ferritin levels is an indicator of monocyte-macrophage activation, a crucial part of the inflammatory storm. Taken together, these results show that patients with an inflammatory storm are more prone to rapid destruction by COVID-19 (25).

### Inflammatory Storm in Diabetic Patients With SARS-CoV-2 Infection

A recently published report, analyzing 138 hospitalized SARS-CoV-2 infected patients, observed that CS-associated neutrophilia triggered by virus invasion and coagulation opening similar to the longer response of inflammation and acute kidney injury has a similar negative impact on SARS-CoV-2, which may be related to the death rate of infected patients (40). A study report that severe SARS-CoV-2 infected patients with pneumonia have a significantly reduced lymphocyte count with high levels of inflammatory factors, especially IL-6 (78). Guo et al. (25) report that, compared with non-DM patients, in DM individuals, the total absolute lymphocyte count in peripheral blood is greatly reduced, and the neutrophil absolute count shows a remarkable increase. Therefore, a high neutrophil–lymphocyte ratio (NLR) in DM patients may increase COVID-19 severity because high NLR is regarded as a poor prognostic factor linked to COVID-19 severity and mortality (109).

## Control of DM Patients with COVID-19

Regarding the management of DM patients with COVID-19, Guo et al. (25) in their study suggest that, whether there is interference or not from other comorbidities, DM patients with viral pneumonia present a more serious status when compared with non-DM ones, stated through organ destruction and inflammatory factor evaluation, that can lead to a complicated prognosis. In the face of such findings, more intensive care should be given to DM patients in case of rapid deterioration. Nevertheless, careful attention should be given to those with nephropathic DM because such patients are at greater risk of viral infection, serious complications, and even death (110, 111).

Current CDC guidance for preventing SARS-CoV-2 infection in DM individuals reveals no differences from the general population, but awareness that DM can lead to a high risk of serious illness should make it easier for healthcare providers to monitor such patients with symptoms of viral infection (i.e., fever and breath shortness) (112). Thus, the role of doctors who give suggestions to DM patients is of crucial importance as they should provide information on extra precaution measures, including social distancing and hand washing toward preventing them from SARS-CoV-2 infection (97). In addition, there should be improved surveillance in DM outpatients for the viral agent, and the starting point for testing this viral infection in such patients should be reduced (64). For instance, Whyte et al. (113) conclude that endothelial dysfunction and microvascular disease may depict as absent a pathophysiological relationship that indicates susceptible populations and may involve specific therapy. Moreover, Zhang et al. (76) postulate that more intensive vigilance and therapy should be considered in DM patients infected with the viral agent, most especially in geriatric ones and in those with preexisting comorbidities. In addition, DM patients are associated with increased mortality and higher disease severity. In a systematic review, Huang et al. (76) show that DM is linked to higher COVID-19 severity, high mortality, disease progression, and development of ARDS.

### Glycemic Control of DM Patients With COVID-19

So far, only a small percentage of experimental studies have addressed the role of hyperglycemia in the respiratory system of individuals with COVID-19 (59). However, studies have reported that hyperglycemia can heighten the glucose concentrations in airway secretions (54). In addition, hyperglycemia can also have a negative impact on pulmonary activity so that respiratory dysfunction triggered by the influenza virus is exacerbated in DM patients (64). DM is related to many changes in lung structure, and these include supplemented vasculature permeability and a relaxed alveolar epithelium in animal models with the disease (114). Glycemic regulation may have a relevant impact on clinical outcomes in individuals with coexistent viral respiratory diseases and DM, such as COVID-19 (65). It has been shown that hyperglycemia and SARS-CoV-2 infection interact in a vicious cycle, in which hyperglycemia induces ACE2 glycation that increases affinity to the SARS-CoV-2. Thus, severe SARS-CoV-2 infection-induced insulin resistance and pancreatic β-cell damage by high levels of pro-inflammatory cytokines can also lead to hyperglycemia (115). Thus, strict control of blood glucose in DM patients with COVID-19 is mandatory to prevent life-threatening complications.

### Control of Hypertension in DM Patients With COVID-19

The coexistence of DM and hypertension requires judicious control of blood pressure increases. A distinct caveat in hypertension therapy in such patients shows that coronaviruses can bind to cells via ACE2, which means that patients who receive therapy with drugs of pharmacological origin that rise ACE2 levels may be put in jeopardy (65). The prevalence of hypertension and CV diseases is clinically relevant in COVID-19 patients, particularly in the elderly with DM. Based on animal models, some concerns have been raised on RAS inhibitors, particularly ARBs, as they can affect ACE2 expression despite there being no clinical evidence that they should be restricted or momentarily obsolete in COVID-19 patients (53). Thus, the European Society of Cardiology (ESC), the American College of Cardiology, the American Heart Association and the Heart Failure Society of America (ACC/AHA-HFSA), and the American Society of Hypertension (ASH) have issued policy statements that strongly recommend DM patients continue therapy with their usual antihypertensive treatment as no empirical reports exist suggesting that therapy with ACE inhibitors or ARBs should be stopped due to SARS-CoV-2 infection. Nonetheless, the issues that cannot be recognized in relation to COVID-19 and DM are significant and numerous (64).

### Control of Neuroendocrine Diseases in COVID-19 Patients With DM

A study by Kaiser et al. (116) found that, in COVID-19 patients, those with pituitary or other neuroendocrine disorders need to be addressed. Patients that have a shortage of primary adrenaline may develop DM insipidus, which further complicate both electrolyte and fluid disorders. Thus, close control and judicious replacement of water and electrolytes to avoid hyponatremia or hypernatremia is crucial to block the high loss of insensitive fluid linked to fever and tachypnea, combined with impaired fluid intake and altered levels of consciousness (117). Indeed, DDP4 inhibitors reduce mortality and COVID-19 severity in DM patients through attenuation of SARS-CoV-2 entry and associated pro-inflammatory cytokine release (118).

### Control of Kidney Diseases in Diabetic Patients With COVID-19

ACEi/ARB therapy use has no have negative impact on COVID-19 morbimortality in synergy with CV disorders (119). D'Marco et al. (72), testing a hypothetical assertion, showed a worsening of DKD that could result in the progression of a serious CKD stage, need for renal replacement, and even death. The authors also postulate that the actual observations support the assertion that reduced immune system defenses and other kidney-associated factors make DM patients more susceptible to infections by certain infectious agents (72).

### Clinical Medication of DM Patients With COVID-19

Recent studies raise the hypothesis that both the treatment course and prognosis of COVID-19 should be grouped, depending on the presence or absence of comorbidities into types A, B, and C. Type A refers to patients with the virus and pneumonia and without comorbidities, type B patients with the virus and pneumonia and comorbidities, and type C represents COVID-19 patients with pneumonia and multiorgan dysfunction (120). In China, Yan et al. (121) studied the clinical features and outcomes in 48 patients with extreme DM and SARS-CoV-2 and compared them with 145 severe COVID-19 patients hospitalized but without DM. As main findings, the authors show that DM patients with serious viral infection had a more severe response due to inflammation and were more prone to require mechanical ventilation and experience death (and survival delay) when compared with non-DM. Regarding clinical medication, the authors state that the insulin dose increased after the patient was infected with SARS-CoV-2, which depicts that the viral agent plays a direct role in glucose metabolism of such patients. Consequently, glucose metabolic dysfunction triggers DM and then impacts pneumonia severity, acting as an amplification loop (25). A study carried out in more than 500 COVID-19 patients revealed that hyperglycemia has often been temporary and usually addressed in most patients after hospital discharge (63). For such hospitalized patients, it is imperative to ensure an excellent glycemic control and look for blood sugar results of 4–10 mmol/L to halt bad results (65, 66). Thus, when reacting to the novel coronavirus pandemic, policies and actions need to involve DM when health inequities are not triggered (49). Indeed, it is reported that uncontrolled or poorly regulated hyperglycemia for any reason, including DM or stress hyperglycemia, is linked to poor outcomes in COVID-19 patients (122). Therefore, strict glucose monitoring and control is advisable in severely affected COVID-19 patients even in non-DM ones (123, 124). For example, in India, the T2DM treatment with hydroxychloroquine has been approved as an alternative for patients with a non-controlled DM with other hypoglycemic agents (i.e., stable-dose insulin therapy with metformin and glimepiride) (125). Moreover, despite inflammation being linked to impaired glucose regulation, the underlying mechanism of the hypoglycemic effect of hydroxychloroquine is still not clear (106).

Micallef et al. (126) indicated that corticosteroid therapy could worsen a patient's condition and that non-steroidal anti-inflammatory drugs, such as ibuprofen, might increase the risk of developing a complicated type of the disease. Indeed, according to the updated clinical guideline, the WHO recommends the use of corticosteroids in severe cases of SARS-CoV-2 infection (127). It is reported that respiratory viral infections are linked to development of DM due to autoimmune pancreatic β-cells injury (128). Specifically, in COVID-19 patients, SARS-CoV-2 may trigger development of DM due to direct pancreatic β-cell injury of SARS-CoV-2 due to a higher expression of ACE2, a receptor for this virus, or indirectly through induction of insulin resistance triggered by SARS-CoV-2-induced oxidative stress (129). Likewise, acute pancreatitis and secondary DM have been reported as an adverse effect of ritonavir and lopinavir, commonly used drugs for the treatment of COVID-19 (130). Likewise, the constant use of ARBs and ACEIs are associated with immunomodulatory properties and reduced pulmonary and systemic inflammatory reactions due to delayed cytokines (131, 132). SARS-CoV-2 may have a direct effect on endothelial cells so that other drugs may also be successful in the treatment of COVID-19 patients via their positive impact on endothelial cells, including α1-adrenergic receptor blockers (e.g., doxazosin) (133), modulators of Sigma receptors (134), metformin (135), indomethacin (136), endothelin receptor antagonists (e.g., bosentan) (137), and heparin and low-molecular weight heparin (LMWH) (138). These drugs, which are often used by DM patients, particularly metformin, reduces endothelial dysfunction and associated complications.

APN01 is a recombinant human ACE2 produced by APEIRON to treat ARDS, pulmonary arterial hypertension, and acute lung injury; briefly, it works by delaying the virus entry and spread and, therefore, may be of high benefit although the clinical trials are still ongoing (100). Indeed, recombinant human ACE2 increases metabolism of vasoconstrictor Ang II to vasodilator Ang1-7, which attenuates development of ARDS (139).

In this sense, the optimal therapy for these patients should derive from an approach involving a consortium of multidisciplinary members, including specialists in infectious diseases, emergency medicine, endocrinology, and respiratory function. Furthermore, backup from exercise rehabilitation specialists and nutritionists may be needed during a longer period of recovery and hospital stay (140). Finally, and regarding therapy, although there are different clinical trials and studies ongoing to assess the efficacy and safety of possible therapeutic alternatives, such as the use of chloroquine phosphate, lopinavir/ritonavir, remdesivir, interferon, tocilizumab, ribavirin, and arbidol, among others (110), no drug against SARS-CoV-2 has been developed and officially approved so far (141).

## Conclusion

In short, DM patients are at a higher risk of developing the severe form of COVID-19 as a consequence of the presence of cardio-metabolic and immunological disorder, including insulin resistance, prolonged hyperglycemia, and hyperinflammation despite other comorbidities, such as hypertension, also maybe boosting the risk. However, and noteworthy, SARS-CoV-2 may cause transient hyperglycemia even in non-DM patients as it is able to induce insulin resistance and/or damage to pancreatic β-cells. Thus, it is essential to ensure strict glucose monitoring in COVID-19 patients with DM to prevent the occurrence of life-threatening complications. Regarding pharmacotherapy in DM, the use of DDP4 inhibitors, mainly sitagliptin, have demonstrated noteworthy anti-inflammatory and anti-SARS-CoV-2 activity, so their use seems to be extremely useful in the management of DM patients with COVID-19.

## Author Contributions

AM, VO, DH, IR, OA, NR-P, AZ-B, MS, HH, NC-M, and GB wrote and revised the paper. All authors have read and agreed to the published version of the manuscript.

## Conflict of Interest

The authors declare that the research was conducted in the absence of any commercial or financial relationships that could be construed as a potential conflict of interest.
